# Autoimmune Glial Fibrillary Acidic Protein Astrocytopathy Associated With Area Postrema Syndrome: A Case Report

**DOI:** 10.3389/fneur.2021.803116

**Published:** 2021-12-24

**Authors:** Xin Gao, Ying Tang, Guo-Dong Yang, Wu Wei

**Affiliations:** Department of Neurology, Jiu Jiang No.1 People's Hospital, Jiujiang, China

**Keywords:** glial fibrillary acidic protein astrocytopathy, area postrema syndrome, case report, autoimmune disease, immunotherapy-responsive

## Abstract

Glial fibrillary acidic protein astrocytopathy is an immunotherapy-responsive autoimmune disease of the central nervous system with various clinical manifestations; among these, there are few reports about area postrema syndrome (APS). The authors present the case of a female patient admitted to the hospital with intractable nausea and vomiting as the predominant symptom. The patient's cerebrospinal fluid was tested by cell-based assays (CBA) and found positive for the presence of anti-glial fibrillary acidic protein (GFAP) antibody, in addition, serological testing showed elevated levels of thyroglobulin and thyroperoxidase-specific antibodies. Brain and cervical MRI showed abnormally high signal on the T2 sequence in the dorsal medulla oblongata and right pontine arm. Therefore, the patient was diagnosed with autoimmune GFAP astrocytopathy. The symptoms improved rapidly after treatment with corticosteroids, and no recurrence has been observed thus far. APS may be a relatively rare clinical manifestation of GFAP astrocytopathy. Importantly, such presentation is challenging to correctly diagnose without typical MRI imaging findings. However, the detection of antibodies in the cerebrospinal fluid or serum may be valuable. Systemic and neurological autoimmunity often coexist, comprehensive antibody screening should be conducted.

## Introduction

Several recent reports have outlined the clinical and pathophysiological characteristics of autoimmune glial fibrillary acidic protein **(**GFAP) astrocytopathy (1–3). Specifically, GFAP-immunoglobulin G (IgG) has been highlighted as fundamental in the diagnosis of the disease, as its presence in the cerebrospinal fluid (CSF) is considered to be a highly specific biomarker. Regardless, the pathogenesis and pathophysiological mechanisms underlying GFAP astrocytopathy have yet to be elucidated. An association to tumors, viral infection, and autoimmune disease have been proposed as possible pathogenic mechanisms (4).

The predominant clinical presentations of the disease are meningoencephalomyelitis and its different forms. Common clinical symptoms include fever, headaches, epilepsy, blurred vision, and ataxia (3), but intractable nausea and vomiting are rarely reported as the predominant symptoms. Here we report the case of a patient with prolonged vomiting and nausea associated with GFAP astrocytopathy.

## Case Description

**Figure 1:**
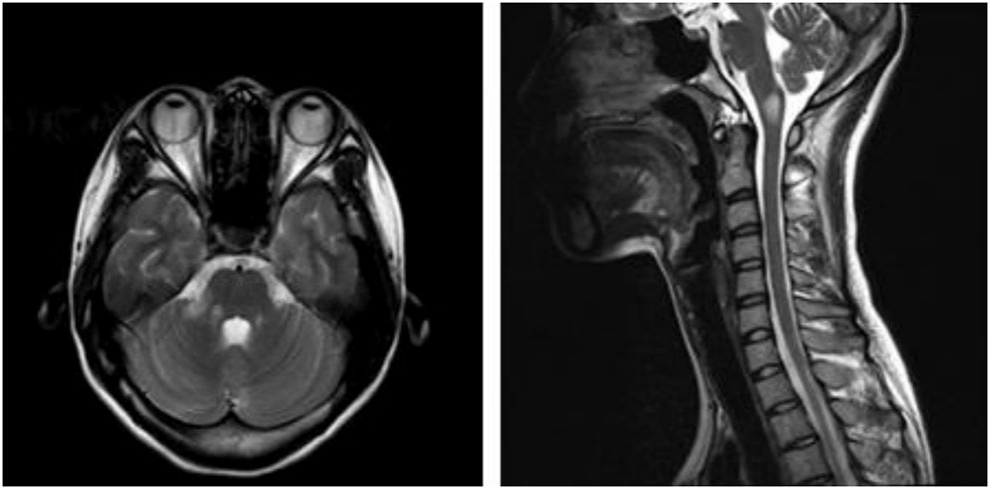
T2-hyperintense lesions in the dorsal medulla oblongata and right middle cerebellar peduncle.

A 21-year-old female patient was admitted to our hospital with complaints of intractable nausea and vomiting, which had been present for the previous 25 days. Medical history was unremarkable. On the 3rd day after onset, she visited a neurologist, who confirmed that CT imaging of the brain showed no abnormalities. During the disease course, the patient also developed dizziness, right facial numbness, and right ear distension with hearing loss, but no improvement was observed after symptomatic treatment. Neurologic examination revealed ataxia, right horizontal gaze-evoked nystagmus, hearing loss in the right ear, and decreasing superficial sensation on the right side of the face. Intracranial pressure measured through lumbar puncture fell within the reference range (135 mmH_2_O; reference range 80–180 mmH_2_O). However, CSF analysis revealed an elevated total white blood cell count of 50 cells/μl (reference range <10 cells/μl) while protein levels were normal (0.42 g/L; reference range <0.45 g/L). The bacterial culture, acid-fast staining, and India Ink Preparation tests were negative. Blood analysis showed high levels of thyroglobulin antibody (947.40 IU/ml; reference range <115 IU/ml) and thyroid peroxidase antibody (93.4 IU/ml; reference range <34 IU/ml). The levels of parathyroid hormone, glycosylated hemoglobin, ceruloplasmin, vitamin B12, thyroid-stimulating hormone (TSH), free T4, T3, rapid plasma reagin (RPR), HIV antibody, and rheumatoid factor were normal. The absence of HIV and hepatitis C virus (HCV) was also confirmed. In addition, the levels of the following autoimmune antibodies were normal: antinuclear, anti-double-stranded DNA, anti-cardiolipin, anti-Smith, anti-Scleroderma-70, Sjögren's syndrome-A, and Sjögren's syndrome-B. Similarly, the levels of antibodies against myelin oligodendrocyte glycoprotein, aquaporin-4 (AQP4), and N-Methyl-D-aspartate (NMDA) receptors antibodies in the serum and CSF were normal. However, a cell-based assay revealed abnormal levels of GFAP antibodies (1:32). Further, imaging revealed an abnormally high signal on T2 sequences in the dorsal medulla oblongata and right middle cerebellar peduncle (Figure 1). The abdominal ultrasound was normal. Therefore, autoimmune GFAP astrocytopathy was diagnosed, and methylprednisolone was administered intravenously (1,000 mg/day for 5 days). Somatosensory, auditory, and visual evoked potentials were normal (evaluated after 3 days of treatment). After 5 days of high-dose corticosteroids, the clinical symptoms significantly improved. Prednisone (60 mg/day) was continued orally and the dosage was decreased within 6 months. No recurrence has been observed thus far.

## Discussion

Autoimmune GFAP astrocytopathy is a recently discovered disease that was first identified in 2016 by Fang and colleagues (Mayo Clinic). Lesions are mainly present in the meninges, brain, spinal cord, and optic nerve, but other areas such as the thalamus, cerebellum, basal ganglia, midbrain, pons, and medulla oblongata are also affected (5). Common clinical symptoms include headaches, seizures, delirium, psychiatric disturbance, and blurred vision. Typically, in patients with autoimmune GFAP astrocytopathy, cranial MRI shows striking radial linear perivascular enhancement in the T2 phase that is perpendicular to the ventricles. In addition, spinal cord MRI shows extensive T2 hyperintensity in the longitudinal direction (often ≥ 3 vertebral segments), and abnormal enhancement on T1 weighted images is observed in two-thirds of patients (2). In addition, neoplasms, which usually develop within 2 years after disease onset, can be found in about 22% of patients, with ovarian teratoma being the most common type (2).

The etiology and pathogenesis of autoimmune GFAP astrocytopathy are still poorly understood. GFAP-IgG, although important for disease diagnosis, is unlikely to be directly pathogenic, as GFAP is intracellularly located in astrocytes (2, 6). In general, coexisting neurological or systemic autoimmunity are relatively common, as shown by the fact that non-neural autoantibodies have been detected in 76% of autoimmune GFAP astrocytopathy patients in a Chinese cohort (5). Our patient specifically presented with higher levels of thyroglobulin and thyroperoxidase-specific antibodies. Consequently, considering the prevalence and adverse effects of autoimmune conditions in the disease (7), comprehensive antibody screening should be conducted. In addition, the symptom improvement observed in our patient after high-dose corticosteroids further solidify the mainstream hypothesis that sensitivity to corticosteroids is a hallmark of the disease (2).

Area postrema syndrome is defined as intractable nausea, vomiting, or hiccups that cannot be explained by other conditions. The diagnosis of this syndrome is performed according to clinical rather than MRI features, as clinical symptoms may not match with imaging findings (8–10). Due to the absence of the blood-brain barrier in this region, the area postrema is particularly susceptible to the infiltration of many proteins and peptides that are usually excluded from other brain tissues. The area postrema is one of the most easily attacked areas by AQP4 antibodies due to the increased presence of astrocytes, which are rich in APQ4 antigens. In recent years, APS has been recognized as one of the core symptoms of neuromyelitis optica spectrum disorder (NMOSD) (11), the involvement of the area postrema, and the presence of clinical APS symptoms are the major features in this disorder (12). A multicenter study showed that, in patients with NMOSD, isolated APS was the first symptom in 7.1–10.3% of cases, while it accompanied other symptoms in up to 60% of individuals. In addition, about 20% of patients presenting with APS had been misdiagnosed as having digestive system diseases (2).

Thus far, only one patient with autoimmune GFAP astrocytopathy has been reported to present with APS as the first symptom (13), suggesting that APS may be a relatively rare clinical manifestation. Specifically, the absence of typical MRI features in our patient, where lesions were mostly confined to the dorsal medulla oblongata, prompted a preliminary diagnosis of NMOSD. Moreover, non-specific viral-like prodromal symptoms, such as chronic diarrhea, nausea, and vomiting, have been shown to be common in patients with autoimmune GFAP astrocytopathy and can mimic APS (14, 15). However, the unresponsiveness to symptomatic therapies in our patient favored an APS over viral-like gastrointestinal prodromes (8).

It is worth noting that both our patient and the individual reported by Ciron et al. were admitted to the neurology department nearly 1 month after symptom onset. In this regard, Kimura et al. suggest that early immunotherapy prior to irreversible injury may reduce the probability of recurrence (3). Accordingly, a follow-up study showed that patients who were diagnosed with autoimmune GFAP astrocytopathy more than 1 year after symptom onset and were treated with corticosteroids and immunoglobulin had a poor prognosis (16). Although this was a small sample study, it suggested that the long timespans that in some cases are necessary to obtain a correct diagnosis result in the poor recovery of daily functions. Therefore, we recommend that GFAP-IgG detection in the CSF be considered for patients who present with intractable nausea and vomiting, especially in those cases where extensive gastroenterological evaluation is negative and MRI findings suggest neurological autoimmune diseases.

There are several limitations in this report. Firstly, a variety of conditions can cause intractable nausea and vomiting, including food poisoning, gastroesophageal reflux disease, and gastroparesis. However, in this case, we did not perform an extensive gastroenterological evaluation to identify any potential digestive system diseases or associated malignancies. Secondly, imaging and serological follow-up tests were not performed due to the rapid improvement of symptoms, thus, it is unknown that whether radiographic changes match with the improvement of clinical symptoms. In conclusion, atypical autoimmune GFAP astrocytopathy with APS could be easily missed or misdiagnosed even by experienced neurologists. Therefore, we recommend that in case disease etiology remains unclear even after detailed examinations and symptomatic treatment is ineffective, autoimmune GFAP astrocytopathy should be considered for patients with intractable nausea and vomiting.

## Data Availability Statement

The original contributions presented in the study are included in the article/supplementary material, further inquiries can be directed to the corresponding author/s.

## Author Contributions

XG performed case information collection, literature review, and drafted the manuscript. YT, G-DY, and WW contributed to the collection of case information. All authors contributed to the manuscript and approved the submitted version.

## Conflict of Interest

The authors declare that the research was conducted in the absence of any commercial or financial relationships that could be construed as a potential conflict of interest.

## Publisher's Note

All claims expressed in this article are solely those of the authors and do not necessarily represent those of their affiliated organizations, or those of the publisher, the editors and the reviewers. Any product that may be evaluated in this article, or claim that may be made by its manufacturer, is not guaranteed or endorsed by the publisher.
